# Workplace Bullying and Coping Strategies Among Portuguese Healthcare Professionals

**DOI:** 10.3390/ijerph22040475

**Published:** 2025-03-23

**Authors:** Ana Isabel Sani, Mariana Magalhães, Rute F. Meneses, Carla Barros

**Affiliations:** 1 Faculty of Human and Social Sciences, University Fernando Pessoa (FCHS-UFP), Praça 9 de Abril, 349, 4249-004 Porto, Portugal; rmeneses@ufp.edu.pt (R.F.M.); cbarros@ufp.edu.pt (C.B.); 2 Research Centre on Child Studies (CIEC), University of Minho (UM), 4710-057 Braga, Portugal; 3 Observatory Permanent Violence and Crime (OPVC), University Fernando Pessoa (UFP), 4249-004 Porto, Portugal; mariana@fpce.up.pt; 4 Research, Innovation and Development Institute of Fernando Pessoa Foundation (FP-I3ID), Praça 9 de Abril, 349, 4249-004 Porto, Portugal; 5 Faculty of Psychology and Educational Sciences, University of Porto (FPCE-UP), 4200-135 Porto, Portugal; 6 Center of Psychology, University of Porto (CPUP), 4200-135 Porto, Portugal; 7 Health Research Network, University Fernando Pessoa (RISE-UFP), 4249-004 Porto, Portugal

**Keywords:** healthcare professionals, coping strategies, workplace bullying

## Abstract

Workplace bullying is a violent, devastating social phenomenon that affects professionals from various fields. The present study aimed to map the bullying behaviors suffered by Portuguese healthcare professionals in the workplace and the coping strategies they used. In this quantitative, cross-sectional, and correlational study, 208 Portuguese healthcare professionals participated by completing an online survey consisting of a sociodemographic questionnaire and two instruments, the Negative Acts Questionnaire—Revised and the Brief COPE. The association between sociodemographic characteristics and victimization, patterns of coping strategy frequency, and the relationship between negative work experiences and coping strategies were analyzed. It was found that only 35 participants self-identified as victims of bullying, and there was no association between victimization and any sociodemographic characteristics. However, it was observed that nurses are more frequently victims of bullying than doctors. Regarding coping strategies, participants on average used more planning and active coping. Moreover, women more frequently used social and emotional support as a coping strategy. Additionally, an association was observed between negative work experiences and coping strategies, with a higher frequency of coping strategies among those who reported more negative experiences. Finally, victims of workplace bullying reported higher use of coping strategies such as venting, distraction, and behavioral disengagement. The results are discussed based on the empirical literature on the topic, and they support reflection on the practical and scientific implications of research on workplace violence, emphasize the potential benefits of our research beyond the individual level, highlight how it could inform policies, improve institutional practices, and foster a healthier work environment for healthcare professionals.

## 1. Introduction

The healthcare sector is, without a doubt, one of the most complex and stressful environments to work in [1]. Its demands—long working hours, high-pressures situations, life or death decisions, strict power chains, etc.—can feel overwhelming and have a significant impact on its employees [2]. Overall, there are many experiences that can result in negative consequences for the employee [3], but some are more impactful than others. Being bullied in the workplace, especially in such a demanding and defiant one, is one of them [2]. At the same time, this phenomenon seems to be promoted by demanding and defiant workplaces [4].

Bullying is an aggressive conduct that a person, of any age group, gender, or profession, may adopt toward someone with the purpose of causing harm in various contexts, such as the workplace [5,6,7]. It distances itself from other violence types by being an intentional, recurrent phenomenon, held within a power-imbalanced relationship [1,2,8,9]. More specifically, workplace bullying is a violent social phenomenon that affects professionals around the world and has been growing in frequency [10,11].

The concept of bullying in the workplace is intricate and multidimensional, encompassing several definitions and terminology that various authors have employed to depict this phenomenon [7], such as harassment [12], mobbing [13], moral harassment [14], or incivility [15]. In this study, we chose to use the concept of workplace bullying, emphasizing the exercise of intentional psychological, verbal, or physical aggressions, and/or power and control over a person, which takes place repeatedly and in a work context, in this case against professionals in institutions that provide healthcare.

Workplace bullying directed at healthcare professionals is a pervasive issue in numerous countries [16,17,18,19]. The prevalence of this phenomenon varies across regions, with European studies highlighting that the variability in prevalence is influenced by differences in definitions and measurement methodologies (behavioral versus self-reporting). In a cross-sectional study involving 205 employees, Cerdeira et al. [20] employed various assessment tools and reported that the prevalence of workplace bullying ranged from 9.96% to 28.8%.

The reviewed literature indicates that the incidence of workplace bullying is often higher in the public sector (e.g., services, health, education, and social assistance) compared to the private sector [21]. Lever et al. [22] conducted a systematic review and reported that the prevalence of bullying among healthcare professionals varies widely, ranging from 3.9% to 86.5%. Furthermore, a study conducted in Portugal by Norton et al. [23] found a prevalence of bullying among healthcare employees to be 8%, with nurses as a group particularly affected.

Multiple studies have consistently highlighted the devastating consequences of workplace bullying on the physical and psychological health of victims [24,25]. These consequences can reveal themselves through clinical symptoms as anxiety, depression, generalized stress, feelings of impotence, decreased self-esteem, lack of interest in the profession, and risk of death [26,27]. Such modifications may make it more difficult for certain healthcare professionals to perform their duties in providing care or making recommendations for medical care [1,2,8,10].

The stress caused in the health professional’s life can be so great that it can also have major repercussions for users and organizations [28]. According to the literature [25,29,30], workplace bullying in healthcare contexts jeopardizes a safety culture, thus posing a serious risk to patient outcomes and to the healthcare professionals’ well-being. Lee et al. [31] conducted a qualitative meta-synthesis of studies on workplace bullying among nurses and found that there are various forms of violence (horizontal and/or vertical, direct and/or indirect) to which these professionals are subjected, accepting and tolerating this violence, often instilled in the very ineffective functioning of their organization. Aiming to identify work-related factors that could influence workplace violence in healthcare environments, Barros et al. [24] developed a study with 276 healthcare professionals (nurses, physicians, healthcare and administrative assistants) and concluded that there is a significant association between psychosocial risk factors (e.g., working hours, work relationships, employment relations, high demands, and work intensity) and vicarious, physical, and psychological violence.

According to previous studies [4,32], several sociodemographic characteristics have been associated with workplace bullying; however, the results were not always coherent. In one Portuguese study that compared Oporto and Azores, age was significantly associated with victimization, as well as having a partner and a definitive employment relationship [4]. Another research pointed out that young, female professionals are at higher risk of suffering bullying in the workplace [32].

In Portugal, workplace bullying is also a current phenomenon with increasing frequency. According to a study carried out during 2022 in one of the two largest metropolitan areas in Portugal, with 24% of national hospitals and 19% of healthcare centers, the majority of reported situations involved verbal violence (followed by moral pressure and discrimination), mainly affecting nurses and doctors, and the majority of victims had already been victimized more than once. Some gender differences were also identified: although statistically significant differences were only observed in terms of verbal victimization, the frequencies were higher for female healthcare professionals compared to their male counterparts. Additionally, it appears that age or marital status do not affect this violent interaction and that professionals who belong to the staff are more vulnerable to violence in the workplace [10]. Nevertheless, not all of these results are consensual in the literature.

Another study conducted in Portugal showed that having a partner and being younger would increase one’s chance of being bullied at their workplace [4]. Antunes et al. [10] did not find a relationship between victimization at the workplace and marital status and reported that professionals under 45 years of age were more likely to be victimized. Moreover, different studies pointed out that this victimization was more frequent among those with a permanent employment relationship [4,10].

In this context, coping strategies emerge as fundamental tools to face this phenomenon in the least impactful way possible. Coping strategies are techniques that individuals use to manage stress, reduce anxiety, and handle difficult emotions or situations [33]. They act as mechanisms to help people maintain emotional and mental well-being (emotion-focused coping) or address the root cause of the stress or problem (problem-focused coping), particularly in the face of adversity. Examples of healthy and constructive (adaptive) mechanisms include defined steps for problem solving and seeking (in)formal support. In contrast, maladaptive mechanisms such as unproductive rumination or self-harm through substance abuse can be unhealthy and potentially harmful. Thus, while some coping mechanisms may provide temporary relief from stress, adaptive coping strategies are more beneficial in the long run, contributing positively to emotional and psychological well-being.

Coping strategies appear to be impacted by contextual factors [34]. Depending on several factors related to the violent interaction—such as the frequency and the severity, among others—the adoption of coping mechanisms may vary (e.g., in type, in frequency). Indeed, situations where workplace bullying is less frequent may be easier to cope with, when compared to frequent and more aggressive bullying situations [2]. Seeking (both instrumental and emotional) support, namely from supervisors, colleagues, and family, seems to be the more common copping strategy [10,35,36,37]. Active/reflective coping [35,37], planning, positive reframing, and acceptance are also frequently reported coping mechanisms, in contrast with substance use [35] and evasive behaviors [35,37]. Also, a study conducted with Portuguese nurses showed that those who had been victims of workplace bullying resorted more frequently to “support seeking”, “evasion” (i.e., behavioral disinvestment), and “substance use” as coping mechanisms, while resorting less to religion and acceptance, when compared to non-victims [37]. Moreover, some gender differences were also spotted: females seem to recur more often to emotional support than males [35]. According to Hawkins and colleagues [36], age and years of experience also influenced people to seek support: younger nurses and those with more years of experience sought social support less frequently.

Considering the above, as well as the lack of studies in Portugal analyzing coping strategies among health professionals, the present research aimed to study the patterns of workplace bullying and coping strategies present among Portuguese healthcare professionals, describing the most common mechanisms and possibly different acts between doctors and nurses. Additionally, we intended to investigate potential differences between people who perceive themselves as victims and self-perceived non-victims with regard to the coping strategies used. Finally, gender differences, any association between adverse experiences in the workplace, and the adoption frequency of different coping strategies were also explored.

## 2. Materials and Methods

### 2.1. Sample

The research sample was collected using a non-probabilistic convenience sampling method, allowing all Portuguese healthcare professionals to participate, and a snowball technique was used among participants. Initially, 276 health professionals participated in this study, including nurses (59.1%), doctors (16.3%), healthcare assistants (13.0%), and administrative assistants (11.6%). Based on this study’s objectives and the materials to be analyzed, only the groups of doctors and nurses were selected for further analysis. Consequently, the sample for the present study consisted of 208 healthcare professionals, aged between 22 and 71, with an average of 39.09 years (*SD* = 10.302). Other descriptive data can be found in Table 1.

As can be seen in Table 1, most participants were female and were married or in a civil partnership. More than two thirds were doctors, and more than half of the participants had up to 15 years of experience, with the majority working in a hospital and in the public sector.

### 2.2. Materials

To characterize the sample collected, items were created a priori to collect information regarding gender, marital status, and profession. Participants were also asked about their length of service, place of work, location of the workplace, sector where they work, and contractual relationship. These items were multiple choice and presented at the beginning of the questionnaire.

The Negative Acts Questionnaire—Revised (NAQ-R) is a widely recognized scale for evaluating workplace harassment and mobbing, originally developed and revised by Einärsen and Hoel in 2001 [38]. The questionnaire comprises 22 items that describe various aggressive and negative behaviors that may occur in the workplace. Respondents assess each item based on its frequency using a five-point Likert scale (from “0” to “4”), ranging from “never” to “daily”. These 22 items can be categorized into three factors: personal bullying, professional bullying, and excessive workload. The Portuguese version of the NAQ-R was validated by Araújo et al. in 2004 [39], which includes one more item (making a total 23 items) that provides a definition of workplace bullying and asks participants whether they perceive themselves as victims. In their study, all factors of the questionnaire showed strong internal consistency, with values of α = 0.89 for personal bullying, α = 0.82 for professional bullying, α = 0.85 for excessive workload, and α = 0.72 overall. Similarly, in the present study, after data collection, strong internal consistency was observed for personal bullying, professional bullying, and excessive workload, with α = 0.94, α = 0.87, α = 0.89, and α = 0.65, respectively, for these categories.

The Brief CODE (Coping Orientation to Problems Experienced) scale, originally developed by Carver et al. [40], is commonly used to evaluate different coping strategies for facing challenges and difficulties. The version of this scale used in this study [41], the Portuguese version validated by Pais-Ribeiro and Rodrigues [42], comprises 28 items regarding both problem-focused (e.g., seeking information) and emotion-focused coping strategies (e.g., avoidance). The items are phrased in terms of the actions that people undertake, and responses are provided on an ordinal scale with four options (from “0” to “3”), ranging from “I never do this” to “I always do this”. The factorial structure of the Portuguese version of the Brief COPE demonstrates characteristics similar to the original scale and confirms the distribution of items across the scales obtained from the factor analysis [42]. The Portuguese validation study showed adequate and satisfactory internal consistency, measured with Cronbach’s alpha, with some items displaying values higher than those of the original scale. Fourteen subscales can be identified in this instrument, namely active coping, planning, using instrumental support, emotional social support, religion, positive reframing, self-blame, acceptance, venting, denial, self-distraction, behavioral disinvestment, substance use, and humor. Information regarding reliability analysis from the original, the validation Portuguese study, and the present study can be found in Table 2.

As can be analyzed, in the present study, every subscale presented reliabilities higher than 0.50. In fact, most subscales presented reliability values above 0.60, except for self-blame and denial. This reliability measure indicates that Brief CORE is also a dependable and lends credibility to this study’s findings.

### 2.3. Ethical Considerations

The present study was submitted for approval to the Ethics Committee of Fernando Pessoa University [Ref. FCHS/PI-219/21-2] by sending in the research protocol to obtain a positive opinion. The protocol included all necessary authorizations by authors for the use of research instruments and adhered to all procedures outlined in the Declaration of Helsinki.

Prior to the construction of the data collection instrument, exhaustive research was carried out to collect scientifically validated scales adapted for the Portuguese population. After selecting the scales that best suited the objectives of the present study, the authors who created them were contacted as part of a request for authorization to use them, and this was granted.

It is important to state that in order to be able to participate in this study, every participant had to give their informed consent before answering its survey. This document clearly outlined the study’s objectives and informed them that their participation was voluntary and that they could withdraw at any time. Furthermore, participants were assured that their responses would remain anonymous.

### 2.4. Procedure

Once a positive opinion from the Ethics Committee was obtained, an online survey was created through the Google Forms platform. The research was widely disseminated to healthcare professionals. The present study was conducted in three hospitals (two from the public sector and one from the private sector) and in two health centers, located in the northern and central regions of Portugal, between March and April 2022.

### 2.5. Data Analysis

The analysis of the data collected within the scope of this research was carried out using IBM SPSS software for Windows, version 28.0. Descriptive statistics procedures were performed to describe the sample obtained. Furthermore, different statistics processes were completed in order to match the objectives of this study, the nature of the data, and the assumptions underlying the statistical techniques. More specifically, the Chi-Square test was used to determine whether there is a significant association between certain categorical variables. Pearson coefficients correlations were used to assess the strength and direction of the linear relationship between continuous variables of NAQ-R and Brief Code, and to reduce the probability of committing a Type I error, we applied Bonferroni correction, setting the *p* level to 0.003 (0.050/18). Finally, an independent-sample *t* test was performed to determine if there is a statistically significant difference between victims and non-victims, males and females, and medical doctors and nurses.

## 3. Results

In order to characterize the sample in terms of the variables under study, descriptive statistics measures were calculated for the two scales and their respective subscales. The results can be seen in Table 3.

An analysis of Table 3 allows us to conclude that the present sample presents low levels of professional bullying, personal bullying, and excessive workload, with the latter condition being the most prevalent. Likewise, it is possible to verify that none of the coping strategies measured is used frequently by the sample, with all average frequencies being less than 2 on a scale of 4 possible points (from “0” to “3”). Even so, within coping strategies, there is a considerable range between the most used—planning and active coping—and the least used—substance use and behavioral disinvestment.

When analyzing the 23rd NAQ-R item, it was possible to understand that the majority of the sample did not identify as a victim of workplace bullying (*n* = 173; 83.2%). However, 16 participants reported being rarely victims of workplace bullying (7.7%), while 15 reported being victimized from time to time (7.2%), 2 several times per week (1%) and 2 almost every day (1%).

Based on this specific item, a new variable was created, separating participants who never had been victims of workplace bullying and those who have. As such, 83.2% of participants were identified as never having been victimized, while 16.83% (*n =* 35) have. Most victims were feminine participants (*n =* 30). In total, 15 had ages between 26 and 35 years old, while 13 were between 36 and 45 years old, 5 between 46 and 54 years old, and 2 between 55 and 64 years old. Also, 30 victims were nurses, while 5 were doctors.

Through the Chi-Square association test, it was possible to understand that being victimized was not associated with the participant’s sex (*p* = 0.807), or with being a medical doctor or a nurse (*p* = 0.367), the time of service (*p* = 0.787), the workplace (*p* = 0.154), the workplace location (*p* = 0.476), a contractual relationship (*p* = 0.987), or the sector in which they work (*p* = 0.514). Also, no differences in age were found between the two groups (*p* = 0.417).

In order to understand whether there is an association between workplace bullying and coping strategies, Pearson correlation coefficients were calculated between the two scales and their respective subscales. The results are presented in Table 4.

In addition to the expected correlations within the measurements of each scale, it was possible to conclude that the scales also present statistically significant, positive correlations between them, albeit very weakly. It was possible to notice that the global index of the NAQ-R scale and its subscales related to professional and personal bullying were correlated with the global index of the Brief COPE scale. Moreover, the NAQ-R global index was correlated with self-blame, denial, and behavioral disinvestment. Also, the professional bullying subscale was correlated with self-blame, venting, denial, and behavioral disinvestment. Regarding the subscale relating to personal bullying, it was only correlated with self-blame. Finally, the excessive workload subscale was correlated only with behavioral disinvestment subscales. Also, the excessive workload subscale was correlated only with the self-blame, denial, and behavioral disinvestment subscales. Finally, the remaining variables did not present a statistically significant correlation between them.

In order to understand if there were any differences concerning coping strategies between participants who were and who were not victimized, independent-sample *t* tests were performed. The results are shown in Table 5.

Victims differ significantly from non-victims concerning venting, self-distraction, and behavioral disinvestment. In fact, victims present higher frequencies concerning these coping strategies, compared to non-victims. Nonetheless, there were no statistically significant differences between the two groups regarding the remaining variables.

Male and female participants were compared regarding coping strategies and harassment experiences through an independent-sample *t* test. The results can be analyzed in Table 6.

As can be seen in Table 6, differences between male and female participants were only statistically significant in the case of the coping strategy “Emotional social support”. In this case, women present a higher frequency than men. Means were statistically similar for the remaining variables.

Medical doctors and nurses were also compared concerning the variables above-mentioned through independent-sample *t* tests. Table 7 presents the results.

Several statistically significant differences were found between the two groups. Nurses present significantly higher frequencies concerning the global index of NAQ-R, professional bullying, personal bullying and religion as a coping mechanism, when compared to medical doctors. No statistically significant differences were found between both groups concerning the other variables.

## 4. Discussion

Workplace bullying is a complex and worrying phenomenon in any context, but it is especially important in such a demanding one as the healthcare sector, mainly due to its personal, social, and labor consequences [1,2,3,4]. As such, this research contributes to mapping the phenomenon in the Portuguese context. Thus, associations with sociodemographic characteristics and victimization by this phenomenon were analyzed, as well as frequency patterns regarding coping strategies and their respective association with victimization.

In a sample where average levels of bullying were low, only 35 participants self-identified as victims. When the experience of victimization among the different sociodemographic characteristics was analyzed, we found that there was no association between them. Additionally, no gender differences were found regarding the global index of negative work experiences, professional bullying, personal bullying, and excessive workload. This result may be seen as somewhat unexpected, as previous studies have shown associations between workplace bullying and gender, age, and marital status [4,10,32]. Nevertheless, the results regarding the relationship between sociodemographic characteristics and these adverse experiences are not consensual in the literature, and the lack of association found in the present study may be due to the reported low frequencies of bullying experiences, which could be due to social desirability bias. Additionally, workplace cultures evolve, and it is possible that the contexts in which our study was conducted reflect shifts in societal attitudes toward gender and bullying. Also, this study’s results should be analyzed with caution, as the majority of its participants were female and nurses, as well as the great majority of the victims. Finally, it was observed that nurses were bullied, professionally and personally, more frequently than medical doctors, which could potentially be explained by the hierarchical environment in the healthcare sector [2], which may be proven to be a psychosocial risk factor associated with particular forms of workplace violence [24].

Regarding coping strategies, results were coherent with previous studies [35,37]: planning and active coping were used more frequently, while behavioral disinvestment and substance use are the least used mechanisms. In this regard, gender differences were found: women used social and emotional support more frequently. Again, these results were equally reported in other studies [37]. Finally, a novel finding was the difference between medical doctors and nurses regarding the frequency with which religious practices are used as a coping strategy, with a higher frequency among nurses.

Contrary to what was observed with regard to sociodemographic characteristics, it was possible to observe that negative experiences in the workplace and some coping strategies are associated with each other. In considering the global indices of the two scales under study, it was possible to verify that a higher frequency of negative experiences in the workplace is associated with a higher frequency in the use of coping strategies. In addition, both the global index of adverse experiences at work and workplace bullying experiences were associated with a higher frequency of self-blame, denial, and behavioral disinvestment, while workplace bullying was also related to venting. Nonetheless, it should be clear that the association between workplace victimization and engaging in these specific coping strategies is weak, which could mean that, although a higher frequency in implementing these strategies tends to occur when there is a higher frequency of workplace bullying victimization, the relationship between these two events may not be that strong. Moreover, when comparing victims and non-victims of professional bullying, it was found that victims had higher frequencies of the following coping strategies: venting, self-distraction, and behavioral disinvestment. The findings described are in line with previous literature that identified an association between workplace bullying and seeking support (e.g., [10,35]) and behavioral disinvestment and substance use [37] as coping strategies.

### 4.1. Limitations

Every study has its limitations. That said, this study’s limitation is related to the sample’s size, which is relatively small and unbalanced, not enabling the inference of causality. Also, it presents a very different number of nurses and medical doctors, and of male and female participants. As such, the obtained results cannot be solidly generalized for the population, and group comparisons are hampered. Moreover, social desirability bias can be a possible methodological limitation of this study, closely related to self-reported measures, as it can lead participants to express in their answers what they think would be considered socially acceptable rather than their honest opinion. Although this is a common limitation among studies in this research field [32], it should be acknowledged, as it can significantly impact the results’ validity and their interpretation, making it even more difficult to generalize the conclusions from this research. Another possible limitation may be related to socioeconomic and cultural differences that perhaps could play their part in the increase or decrease in workplace bullying against healthcare professionals, variables that could not be achieved in this study.

### 4.2. Practical Implications

There are several practical applications that can arise from this study. Contributing to the mapping of the phenomenon in Portuguese territory, the present study provides significant information for the construction of projects and programs that aim not only to reduce or eradicate the phenomenon of bullying in the workplace but also to promote coping strategies to deal with conflict and stress in a workplace as challenging as the healthcare sector.

Additionally, this study paves the way for future studies, preferably with a larger and more representative sample of the population. In this sense, it is also important to recognize that studies such as this one contributes to the visibility of this social phenomenon, both for the community, in general, and for the victims, in particular. In this way, it promotes the identification of this phenomenon in practice—an essential step for intervention.

With awareness of this problem having implications not only for the life of the healthcare professional but also for their users and the organization itself [25,28,29,30], the institution in which these professionals work could implement programs and workshops that focus on building healthy coping mechanisms for employees who are going through negative experiences in the workplace. To this end, it may be relevant for organizations to be open to the internal study of the phenomenon, conducting regular employee surveys to assess the workplace culture and detect early signs of bullying, and if it happens, our findings can be used to adjust policies and interventions as needed. Organizations could be available to flag cases (e.g., using digital platforms to set up confidential reporting channels where employees can safely report incidents of bullying without fear of retaliation, or hiring a third-party service to objectively deal with complaints). It is also suggested that managers and supervisors in organizations receive training on how to intervene in workplace bullying situations, providing proactive leadership that promotes respect and teamwork, preventing future situations. This is only possible in an open and supportive working environment that assumes the principle of ‘zero tolerance’, which guarantees that all reported cases will be investigated promptly with fair and transparent procedures and that actions will be taken to hold aggressors accountable. Organizations can also offer a peer support network for creating ‘Safe Spaces’ for discussions and emotional support or counseling services. Multicomponent interventions involving all stakeholders have shown more positive results compared to isolated interventions; in fact, a holistic approach ensures that bullying is addressed at both individual and systemic levels, leading to more sustainable and effective results [43].

Therefore, increased awareness of working circumstances would enable the creation of occupational health interventions and improved organizational support, which would reduce violence and hostility in the workplace. Any organization can act preventively if it is able to promote a culture of open communication and conflict resolution in the workplace to solve problems before they escalate into bullying situations.

### 4.3. Suggestions for Future Studies

To obtain results closer to the general population, future studies could be carried out using a representative sample. In considering the importance of coping strategies in mitigating the impact of violence, it will also be important to analyze the coping strategies used in a study conducted with a larger, representative sample of the population, explore various coping strategies in detail, including both adaptive and maladaptive strategies, and assess their effectiveness in different contexts and among diverse groups of healthcare professionals.

In addition, future studies should also analyze the mediating role of different coping strategies in the relationship between workplace bullying and its impacts. Furthermore, investigating how intersectional factors such as race, ethnicity, age, and sexual orientation interact with experiences of workplace bullying could contribute to creating inclusive and equitable work environments.

It would be important to collect testimonies from victims of bullying in this labor sector, using qualitative methodologies, conducting in-depth analyses, and then comparing the testimonies of medical professionals and nurses. Implement mixed-method designs that combine quantitative surveys with qualitative interviews or focus groups could provide richer, more nuanced data on the complexities of workplace bullying, including emotional impacts and coping strategies.

Our study was cross-sectional research. However, longitudinal studies to assess how experiences of workplace bullying and coping mechanisms evolve over time may allow for a better understanding of the long-term effects of bullying on healthcare professionals and the potential development of resilience.

The present study focused solely on the victims’ perspectives. However, victims are not the only social actor in this phenomenon; as such, it is extremely important to consider both the perpetrators’ and witnesses’ experiences also. Thus, future studies could focus on studying workplace bullying and its influencing factors from these different points of view. Studies could explore predictors of victimization and the perpetration of workplace bullying in the health sector; for example, the stressful work environment, the organizational culture, the responsibilities covered by each worker, the level of turnover in the team, and the environment among team members, among others, could be considered.

Future studies may explore workplace bullying across different cultural and geographical contexts examining how cultural values and norms influence bullying behavior and responses could provide insights into tailored interventions.

## 5. Conclusions

Considering the relevance of studying an impactful, social phenomenon that requires urgent intervention, the present study is important on several levels. Even with relatively low levels of professional bullying (and potentially higher in larger samples), this research brings to light the existence of victimization experiences that need to be recognized, better known, and intervened. The identification of coping strategies can translate into insights regarding the reality of a population and, thus, help entities, organizations and the working classes in developing valuable support strategies in order to reduce the frequency and impact of this violent scourge. It is also important to note that the results obtained may represent the starting point for future research, thus promoting knowledge in this area—essential for the design of public policies and intervention programs adapted to the healthcare sector.

It is imperative to reiterate the importance of this study for professional practice in the context of health, not only because it brings visibility to an extremely important topic but also because it provides solid ground for the development of prevention policies (e.g., the creation of specific policies and protocols to prevent bullying in the workplace) and awareness-raising and training actions focused on identifying the phenomenon and ways to intervene in the face of it. Promoting an organizational culture that values diversity and cooperation among its employees, regardless of professional status, sociodemographic characteristics, and other potentially differentiating factors, can serve as a protective factor against violent phenomena such as workplace bullying, while it is equally crucial that the work environment itself conveys a clear message of zero tolerance for this type of behavior. This study also highlights the need to promote psychological support resources for workers involved in this phenomenon, promoting their mental health and, consequently, a positive impact on their work and the quality of care they provide within it.

By addressing these critical issues, our research aimed to contribute to the possible influence of the social and institutional frameworks of the Portuguese healthcare system. Understanding the dynamics of workplace bullying will not only help identify the unique challenges faced by healthcare professionals but also inform the development of targeted interventions to improve workplace conditions. Albeit modestly, the insights gained from this study can guide policymakers and healthcare administrators in creating supportive environments that prioritize mental health and well-being, which could ultimately lead to higher job satisfaction and retention rates among healthcare professionals. In addition, identifying effective strategies for dealing with workplace bullying can give professionals the tools to cope with adversity, promoting a more resilient workforce. Taken together, this research seeks to contribute to a cultural change in healthcare institutions, promoting a climate of respect, collaboration, and effective communication that benefits not only the professionals but also the patients they serve.

## Figures and Tables

**Table 1:** Descriptive statistics regarding sociodemographic characteristics.

	*n*	%
Sex		
Feminine	173	83.2
Masculine	35	16.8
Marital Status		
Married/Civil partnership	118	56.7
Single	74	35.6
Divorced/Separated	13	6.3
Widowed	3	1.4
Profession:		
Nurse	163	78.4
Medical doctor	45	21.6
Time of service		
Less than a year	7	3.4
Between 1 and 5 years	40	19.2
Between 6 and 10 years	26	12.5
Between 11 and 15 years	52	25.0
Between 16 and 20 years	28	13.5
Between 21 and 25 years	23	11.1
Between 26 and 30 years	14	6.7
Between 31 and 35 years	8	3.8
More than 35 years	10	4.8
Workplace		
Hospital	137	65.9
Healthcare center	50	24
Clinical practice	21	10.1
Workplace location		
Porto	102	49
Braga	16	7.7
Vila Real	49	23.6
Viana do Castelo	7	3.4
Aveiro	7	3.4
Lisboa	11	5.3
Setúbal	9	4.3
Santarém	1	0.5
Azores	4	1.9
Madeira	1	0.5
Leiria	1	0.5
In which sector do you work?		
Public sector	166	79.8
Private sector	31	14.9
Both	11	5.3
Contractual relationship		
Open-ended contract	172	82.7
Fixed-termed contract	25	12
Temporary contract/Independent employee	11	5.3

**Table 2:** Reliability analysis regarding Brief CODE scale (Cronbach’s alpha).

	Original Study [40]	Validation Study [41]	Present Study
Global index			0.91
Active coping	0.68	0.65	0.78
Planning	0.73	0.70	0.84
Instrumental support	0.64	0.81	0.77
Emotional social support	0.71	0.79	0.81
Religion	0.82	0.80	0.88
Positive reframing	0.64	0.74	0.77
Self-blame	0.69	0.62	0.51
Acceptance	0.57	0.55	0.68
Venting	0.50	0.84	0.75
Denial	0.54	0.72	0.57
Self-distraction	0.71	0.67	0.66
Behavioral disinvestment	0.65	0.78	0.68
Substance use	0.90	0.81	0.78
Humor	0.73	0.83	0.87

**Table 3:** Descriptive statistics from NAQ-R and Brief Code scales and subscales.

	Min.	Max.	Mean	Standard Deviation
NAQ-R				
Global index	0	3.82	0.58	0.63
Professional bullying	0	3.90	0.63	0.69
Personal bullying	0	4.00	0.44	0.60
Excessive workload	0	4.00	0.89	1.05
Brief COPE				
Global index	0	2.46	1.31	0.46
Active coping	0	3.00	1.79	0.74
Planning	0	3.00	1.87	0.73
Instrumental support	0	3.00	1.57	0.82
Emotional social support	0	3.00	1.57	0.86
Religion	0	3.00	0.95	0.91
Positive reframing	0	3.00	1.74	0.77
Self-blame	0	3.00	1.35	0.70
Acceptance	0	3.00	1.74	0.71
Venting	0	3.00	1.52	0.77
Denial	0	2.50	0.82	0.72
Self-distraction	0	3.00	1.42	0.75
Behavioral disinvestment	0	3.00	0.49	0.58
Substance use	0	2.00	0.15	0.39
Humor	0	3.00	1.38	0.81

**Table 4:** Pearson correlation coefficients of NAQ-R and Brief Code scales and subscales.

	1	2	3	4	5	6	7	8	9	10	11	12	13	14	15	16	17	18	19
NAQ-R																			
1. Global index	1	-	-	-	-	-	-	-	-	-	-	-	-	-	-	-	-	-	-
2. Professional bullying	0.95 **	1	-	-	-	-	-	-	-	-	-	-	-	-	-	-	-	-	-
3. Personal bullying	0.88 **	0.74 **	1	-	-	-	-	-	-	-	-	-	-	-	-	-	-	-	-
4. Excessive workload	0.71 **	0.61 **	0.50 **	1	-	-	-	-	-	-	-	-	-	-	-	-	-	-	-
Brief COPE																			
5. Global index	0.22 **	0.22 **	0.22 **	0.08	1	-	-	-	-	-	-	-	-	-	-	-	-	-	-
6. Active coping	0.03	0.02	0.09	−0.10	0.75 **	1	-	-	-	-	-	-	-	-	-	-	-	-	-
7. Planning	0.12	0.15	0.11	−0.03	0.82 **	0.67 **	1	-	-	-	-	-	-	-	-	-	-	-	-
8. Instrumental support	0.15	0.16	0.14	0.01	0.80 **	0.58 **	0.70 **	1	-	-	-	-	-	-	-	-	-	-	-
9. Emotional social support	0.14	0.17	0.11	0.02	0.72 **	0.48 **	0.58 **	0.74 **	1	-	-	-	-	-	-	-	-	-	-
10. Religion	0.14	0.14	0.12	0.09	0.51 **	0.29 **	0.29 **	0.39 **	0.31 **	1	-	-	-	-	-	-	-	-	-
11. Positive reframing	0.04	0.01	0.14	−0.12	0.80 **	0.69 **	0.73 **	0.61 **	0.54 **	0.33 *	1	-	-	-	-	-	-	-	-
12. Self-blame	0.25 **	0.22 *	0.27 **	0.15	0.48 **	0.269 **	0.41 **	0.21	0.17	0.16	0.32 *	1	-	-	-	-	-	-	-
13. Acceptance	0.03	0.04	0	0.03	0.72 **	0.48 **	0.64 **	0.60 **	0.45 **	0.36 **	0.61 **	0.31 **	1	-	-	-	-	-	-
14. Venting	0.20	0.20	0.12	0.12	0.68 **	0.49 **	0.55 **	0.57 **	0.48 **	0.31 **	0.51 **	0.20 *	0.40 **	1	-	-	-	-	-
15. Denial	0.24 **	0.23 *	0.23 **	0.16	0.52 **	0.32 **	0.31 **	0.31 **	0.26 **	0.20	0.29 **	0.28 **	0.19	0.31 **	1	-	-	-	-
16. Self-distraction	0.17	0.19	0.14	0.09	0.63 **	0.36 **	0.47 **	0.40 **	0.49 **	0.20 **	0.39 **	0.27 **	0.41 **	0.38 **	0.32 **	1	-	-	-
17. Behavioral disinvestment	0.26 **	0.24 **	0.20	0.27 **	0.15 *	−0.11	−0.03	−0.02	−0.01	−0.04	−0.06	0.20 **	0.01	0.09	0.24 **	0.15 **	1	-	-
18. Substance use	0.22 **	0.19 **	0.18 **	0.20	0.15 *	−0.04	−0.03	0	0.07	0.07	−0.03	0.17	−0.01	0.01	0.23 **	0.10	0.24 **	1	-
19. Humor	0.02	0	0.09	−0.07	0.65 **	0.50 **	0.46 **	0.44 **	0.33 **	0.25 **	0.64 **	0.20	0.50 **	0.35 **	0.30 *	0.36 **	0.02	0.02	1

* *p* < 0.003 (*p* < 0.050 with Bonferroni correction for 18 tests); ** *p* < 0.001.

**Table 5:** Independent-sample *t* test comparing victimized and non-victimized participants regarding coping strategies.

	Victims (*n* = 35)		Non-Victims (*n* = 173)		*t*	*df*	*p*	*Cohen’s d*
	Mean	Std.	Mean	Std.				
Brief COPE								
Global index	1.45	0.34	1.28	0.47	−1.94	206	0.054	−0.36
Active coping	1.74	0.65	1.80	0.76	0.44	206	0.659	0.08
Planning	2.03	0.71	1.84	0.73	−1.39	206	0.165	−0.26
Instrumental support	1.66	0.75	1.55	0.83	−0.69	206	0.488	−0.13
Emotional social support	1.81	0.78	1.52	0.87	−1.90	206	0.059	−0.35
Religion	1.00	0.85	0.94	0.92	−0.38	206	0.706	−0.07
Positive reframing	1.74	0.76	1.74	0.77	−0.04	206	0.967	−0.01
Self-blame	1.32	0.69	1.51	0.72	−1.52	206	0.129	−0.28
Acceptance	1.76	0.75	1.74	0.70	−0.18	206	0.861	−0.03
Venting	1.89	0.61	1.45	0.78	−3.14	206	0.002 *	−0.58
Denial	0.96	0.75	0.80	0.71	−1.22	206	0.224	−0.23
Self-distraction	1.70	0.73	1.36	0.75	−2.43	206	0.016 *	−0.45
Behavioral disinvestment	0.77	0.57	0.43	0.57	−3.19	206	0.002 **	−0.59
Substance use	0.31	0.61	0.11	0.32	−1.91	37.89	0.064	−0.53
Humor	1.36	0.85	1.38	0.80	0.18	206	0.856	0.03

* *p* < 0.05; ** *p* < 0.01.

**Table 6:** Independent-sample *t* test comparing male and female participants.

	Male (*n* = 35)		Female (*n* = 173)		*t*	*df*	*p*	*Cohen’s d*
	Mean	Std.	Mean	Std.				
NAQ-R								
Global index	0.76	0.88	0.55	0.56	−1.81	206	0.071	−0.34
Professional bullying	0.80	0.88	0.59	0.65	−1.64	206	0.102	−0.31
Personal bullying	0.63	0.93	0.41	0.51	−1.37	38.27	0.178	−0.37
Excessive workload	1.03	1.22	0.86	1.02	−0.86	206	0.453	−0.16
Brief COPE								
Global index	1.25	0.43	1.32	0.46	0.90	206	0.37	0.17
Active coping	1.74	0.61	1.80	0.76	0.44	206	0.659	0.08
Planning	1.91	0.74	1.86	0.73	−0.37	206	0.711	−0.07
Instrumental support	1.43	0.89	1.60	0.80	1.12	206	0.263	0.21
Emotional social support	1.29	0.81	1.62	0.86	2.13	206	0.034 *	0.40
Religion	0.77	0.84	0.98	0.92	1.26	206	0.209	0.23
Positive reframing	1.64	0.71	1.76	0.78	0.80	206	0.423	−149.00
Self-blame	1.33	0.84	1.36	0.67	0.21	206	0.836	0.04
Acceptance	1.63	0.60	1.76	0.73	1.00	206	0.317	0.19
Venting	1.37	0.87	1.55	0.75	1.24	206	0.216	0.23
Denial	0.80	0.77	0.83	0.71	0.20	206	0.842	0.04
Self-distraction	1.41	0.72	1.42	0.76	0.06	206	0.956	0.01
Behavioral disinvestment	0.51	0.69	0.49	0.56	−0.27	206	0.791	−0.05
Substance use	0.14	0.45	0.15	0.38	0.06	206	0.95	0.01
Humor	1.49	0.73	1.49	0.73	−0.85	206	0.395	−0.16

* *p* < 0.05.

**Table 7:** Independent-sample *t* test comparing medical doctors and nurses.

	Medical Doctors (*n* = 45)		Nurses (*n* = 163)		*t*	*df*	*p*	*Cohen’s d*
	Mean	Std.	Mean	Std.				
NAQ-R								
Global index	0.39	0.40	0.64	0.67	3.08	117.49	0.003 **	0.40
Professional bullying	0.39	0.47	0.69	0.73	3.31	108.24	0.001 **	0.44
Personal bullying	0.23	0.30	0.51	0.65	4.11	158.20	<0.001 ***	0.47
Excessive workload	1.01	1.15	0.86	1.03	−0.82	206	0.382	−0.15
Brief COPE								
Global index	1.22	0.52	1.34	0.43	1.60	206	0.110	0.27
Active coping	1.73	0.82	1.81	0.72	0.61	206	0.540	0.10
Planning	1.84	0.87	1.88	0.69	0.26	60.27	0.798	0.05
Instrumental support	1.46	0.93	1.60	0.78	1.06	206	0.290	0.18
Emotional social support	1.36	0.87	1.62	0.85	1.86	206	0.064	0.31
Religion	0.69	0.82	1.02	0.92	2.18	206	0.030 *	0.37
Positive reframing	1.60	0.88	1.78	0.73	1.23	61.63	0.225	0.23
Self-blame	1.24	0.75	1.38	0.68	1.16	206	0.249	0.20
Acceptance	1.72	0.73	1.74	0.70	0.17	206	0.866	0.03
Venting	1.32	0.78	1.57	0.76	1.94	206	0.053	0.33
Denial	0.86	0.85	0.81	0.68	−0.31	60.43	0.757	−0.06
Self-distraction	1.44	0.83	1.41	0.73	−0.24	206	0.812	−0.04
Behavioral disinvestment	0.41	0.58	0.51	0.59	1.03	206	0.304	0.17
Substance use	0.09	0.29	0.16	0.41	1.37	99.13	0.173	0.19
Humor	1.24	0.74	1.42	0.82	1.27	206	0.204	0.22

* *p* < 0.05; ** *p* < 0.01; *** *p* < 0.001.

## Data Availability

The data presented in this article are not readily available since they were not approved to be shared outside of the research team. Requests to access the datasets should be directed to authors.
